# Colored and fluorescent nanofibrous silk as a physically transient chemosensor and vitamin deliverer

**DOI:** 10.1038/s41598-017-05842-8

**Published:** 2017-07-14

**Authors:** Kyungtaek Min, Sookyoung Kim, Chang Gun Kim, Sunghwan Kim

**Affiliations:** 1 Department of Energy Systems Research, Ajou University, Suwon, 16499 Republic of Korea; 2 Immune-Network Pioneer Research Center, School of Medicine, Ajou University, Suwon, 16499 Republic of Korea; 3 Department of Physics, Ajou University, Suwon, 16499 Republic of Korea

## Abstract

Biodegradable and physically transient optics represent an emerging paradigm in healthcare devices by harnessing optically active system and obviating issues with chronic uses. Light emitting components that can efficiently interact with their environments have advantages of high sensitivity, visibility, and wireless operation. Here, we report a novel combination of silk biopolymer and optically active organic dyes resulting in versatile fluorescent silk nanofibers (FSNs). FSNs generated by the electrospinning method exhibit attractive functions of the doped organic dyes along with programming the system that physically disappear at prescribed time. Red-green-blue (RGB) fluorescent nanofibrous mats, eco-friendly and transient fluorescent chemosensors for acid vapor detection, and disposable membranes for nutrition delivery were successfully demonstrated using FSNs. These functions introduced using four water soluble dyes: rhodamine B, sodium fluorescein, stilbene 420, and riboflavin. The FSN with sodium fluorescein especially, showed a sensing capability for hazardous and volatile hydrochloric acid vapors. Delivering riboflavin (vitamin B_2_, an important nutrient for skin care) in the FSN to a biological tissue could be observed by tracing the fluorescence of riboflavin.

## Introduction

One dimensional polymer nanofibers and their networks feature extremely high surface-to-volume ratios, porosity, and permeability that are of interest in sensing^1–3^, tissue engineering^4–6^, drug delivering^7–9^, protective clothing^10, 11^, and filtering applications^12, 13^. Electrospinning is a versatile technique for generating polymeric nanofibers from a broad range of polymers^14, 15^. It provides low-cost and high throughput fabrication as well as the means to control the material traits of polymeric nanofibers such as stiffness, strength, fiber diameter, and porosity. In material aspects of polymeric nanofibers, the growing demand for healthcare looks toward the use of biopolymers including polysaccharides (cellulose, chitin, chitosan, etc.)^16–18^, proteins (silk, collagen, gelatin, etc.)^19–21^, and DNA^22, 23^ due to sustainability, eco-friendliness, and renewable nature based on their inherent properties such as biocompatibility and biodegradability.

In biological and biomedical sciences, nano-optics is playing a remarkable role in imaging, sensing, and therapy. Therefore, the inclusion of optically functionalities to biopolymeric nanofibers can bring together all favorable traits of nano-optics and biopolymeric nanofibers. To date, a variety of optically functional dopants including fluorescent dyes^24–26^, quantum dots^27–29^, and metal nanoparticles^30, 31^ have been employed to tailor optical properties of the host polymeric nanofibers. When all aspects are considered, one will find that silk fibroin from the *Bombyx mori* cocoon is most suitable for the host to demonstrate optically functional biopolymer nanofibers^20^. Biologically and optically active dopants such as enzymes, drugs, and dyes can be stably preserved in the silk matrix under all-water based and mild process^32^. In addition, the silk matrix shows optical transparency and can be easily nanostructured^33^. The favorable material traits of silk fibroin, including biocompatibility, biodegradability, and renewability, make it well suited to serve as a platform for physical transient and water-disposable nano-optics^34, 35^ that can be potentially used near or in the human body.

We report herein the demonstration of electrospun fluorescent silk nanofibers (FSNs) and their applications as a highly sensitive chemosensor and a nutrient deliverer with a physically transient form. The functionalities of FSNs depend on organic dyes doped in the silk solution. High-quality silk FSNs with spatial homogeneities in diameter and fluorescent emissions were obtained for four different water-soluble organic dyes: stilbene 420, riboflavin (vitamin B_2_), sodium fluorescein, and rhodamine B. Among these, the FSN doped with sodium fluorescein could be applied as a highly sensitive fluorescent chemosensor for acid vapors in air. FSN sensors responded to hydrochloric acid (HCl) vapor immediately at lethal concentrations (>300 ppm) and could also detect HCl vapors at low concentrations (~5 ppm). In addition, FSNs could disappear at prescribed time by controlling the crystallinity of the silk fibers. We presented an example of usage of the fluorescent nanofiber mat as a water-disposable skin-type patch or indicator to be attached to clothing and protective equipment. Further, a drug delivery model using silk nanofiber membranes doped with riboflavin (vitamin B_2_) was also suggested. The decrease in fluorescence of nanofiber mats was measured to estimate the amount of riboflavin delivered to the skin.

## Results and Discussions

**Figure 1:**
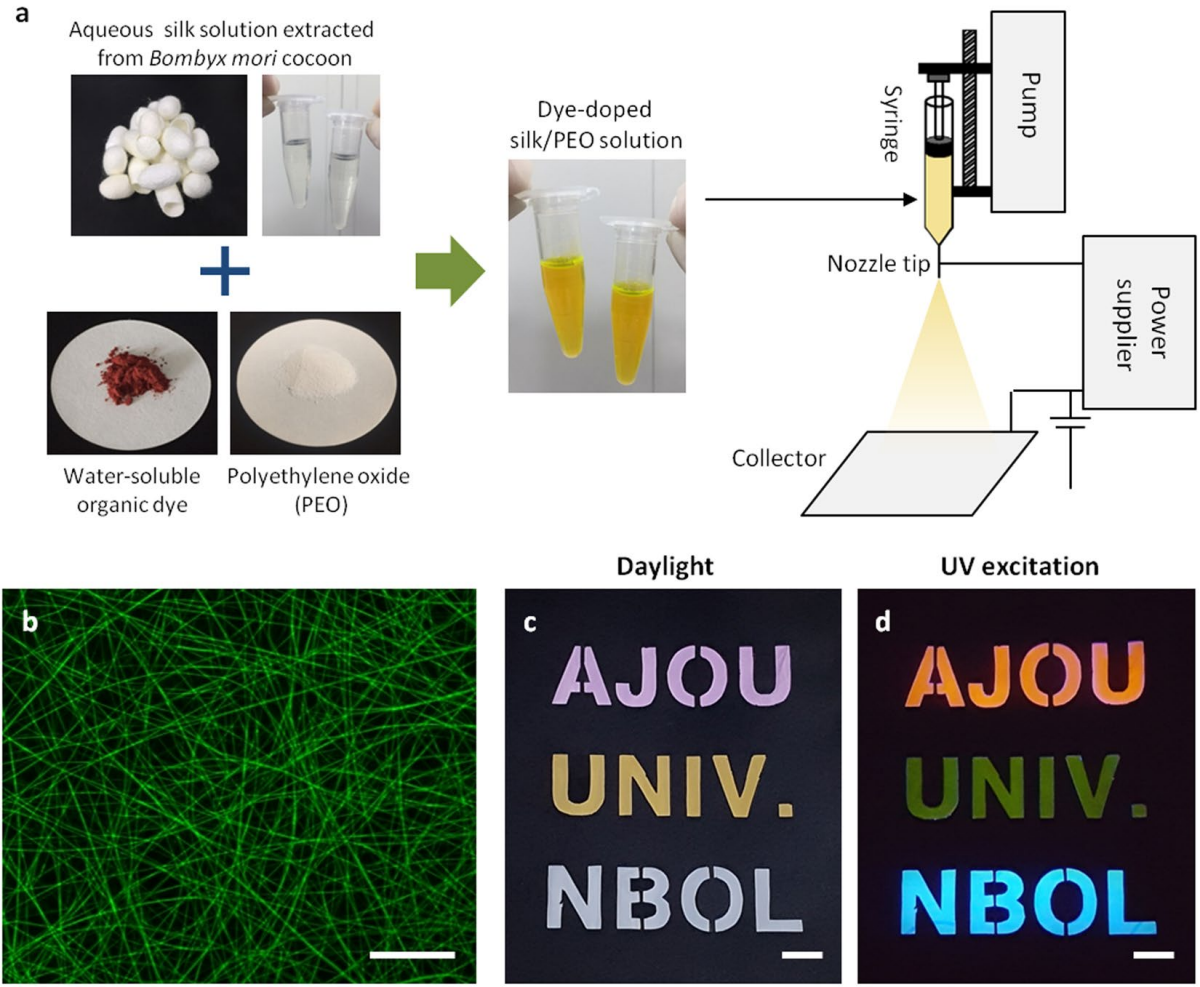
Fabrication of electrospun FSNs. (**a**) Schematic diagram of electrospinning process for the dye-doped FSNs. (**b**) Fluorescent microscope image of the sodium fluorescein-doped FSNs. Character expression using the FSN mats and a shadow mask, representing AJOU UNIV. NBOL (**c**) in daylight and (**d**) under UV excitation (325 nm). RGB fluorescent colors are implemented. Scale bars represent 20 μm for (**b**) and 1 cm for (**c**) and (**d**).

The electrospinning process started from preparing silk bio-ink blended with water-soluble fluorescent dye and polyethylene oxide (PEO) as shown in Fig. 1a. PEO was used to prevent embrittlement of silk nanofibers due to the formation of water-insoluble *β*-sheet^20^. To yield FSNs, the silk ink was injected through a steel capillary tube and then electrically charged with different electric potentials between the capillary tube and the bottom metal collector. As the injected silk ink-jet dried in air, the jet was elongated by electrostatic repulsion in the whipping process. Finally, the elongated silk fiber was collected on a metal plate. We used a commercial aluminum foil for easy separation of silk nanofibers from the foil as well as to keep the cost low. A FSN mat piece with the 1 × 1 cm^2^ size can be prepared at a cost of under US ten cents. The low production cost is important to apply the FSN as a disposable sensor.

To investigate physical and optical qualities of the generated FSNs, fluorescent images, scanning electron microscopy (SEM) images, and photoluminescence spectra were obtained. As shown in Fig. 1b, the FSN blended with sodium fluorescein having a fluorescence maximum at 535 nm shows homogeneous green emission from uniform fibers. This indicates that the dye molecules were uniformly distributed in the silk matrix after the electrospinning process. In principle, any water-soluble dyes can be adapted to generate FSNs. Along with sodium fluorescein, stilbene 420, riboflavin (vitamin B_2_), and rhodamine B were successfully used to generate FSNs. Supplementary Fig. S1 shows that all FSNs have good uniformity in diameters and shows fluorescence agreeing with intrinsic properties of each dyes. The FSN mat could be used as a color converter, similar to phosphors, for white light emitting diodes (LEDs). Figure 1c shows three kinds of FSNs, which were doped with rhodamine B, sodium fluorescein, and stilbene 420, for spelling “AJOU”, “UNIV.”, and “NBOL”, respectively (from top to bottom). To express the letters, the FSN mats were placed behind a shadow mask using a black paper. Under daylight irradiation, FSNs exhibit pink, yellow, and white colors, respectively. Under excitation using the 325-nm He-Cd laser, homogeneous fluorescence in orange-red (the wavelength of the fluorescence peak, λ_peak_ ~ 585 nm), green (λ_peak_ ~ 535 nm), and blue (λ_peak_ ~ 435 nm) could be observed (Fig. 1d). The versatility of the electrospinning process would help in adding engineering optical functionalities as well as generating low-cost and large area nanostructured biopolymer networks.

**Figure 2:**
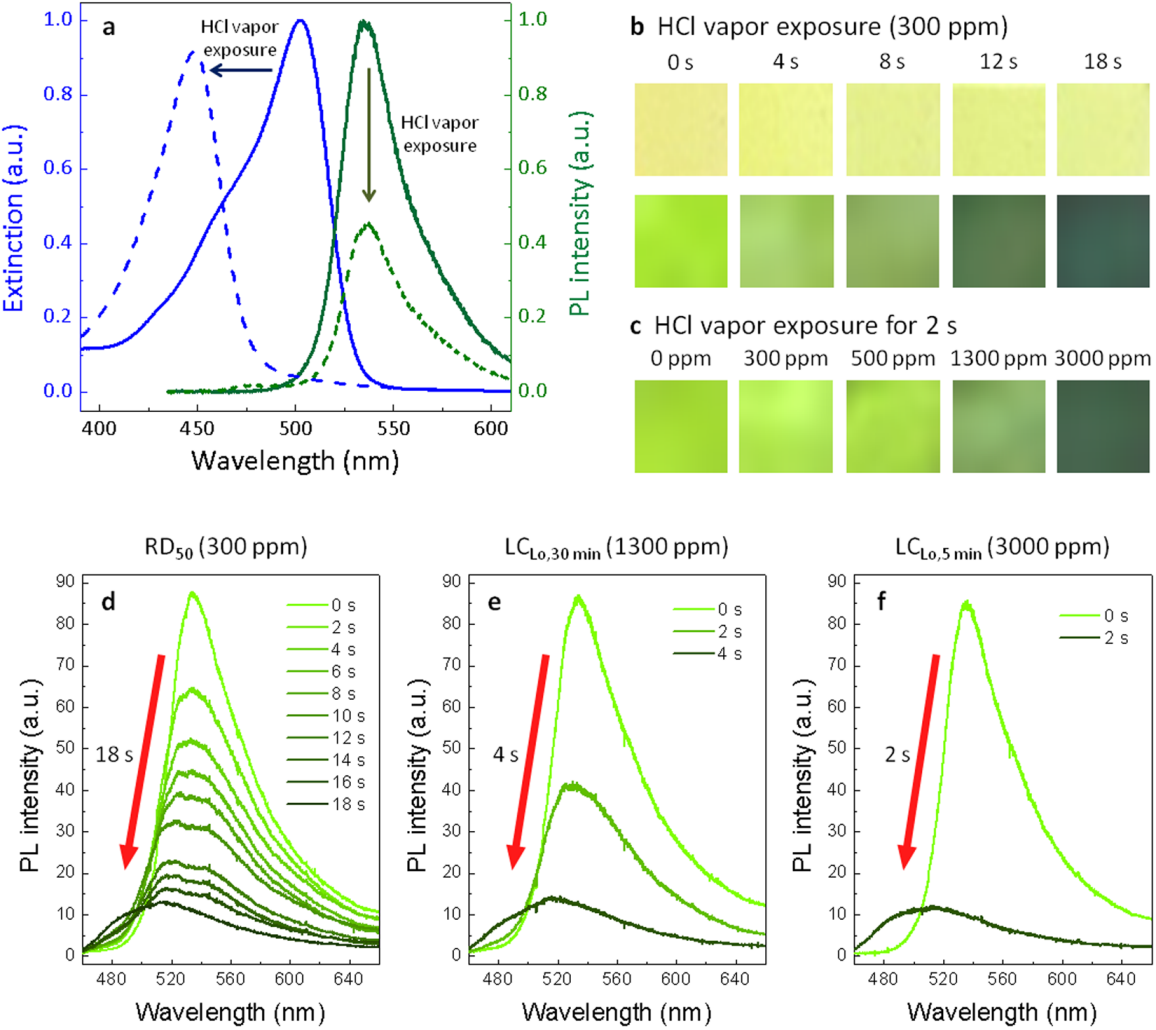
Results of the HCl vapor sensing experiment at lethal concentrations. (**a**) Absorption (blue line) and emission (green line) spectra of the sodium fluorescein-doped silk film before (solid) and after (dotted) exposure to HCl fume. (**b**) Time-dependent changes of external color and fluorescence of the FSNs exposed to 300 ppm HCl vapor. (**c**) Concentration-dependent fluorescence changes of FSNs exposed to HCl vapor for 2 seconds. Changes of photoluminescence spectra and response times of the FSN sensors in (**d**) 300 ppm, (**e**) 1300 ppm, and (**f**) 3000 ppm.

The high surface-to-volume ratio of FSN mats is advantageous for utilizing colorimetric and fluorescent gas sensors. HCl vapor is chosen as an analyte since it is widely used to produce organic compounds but is corrosive and harmful for humans. The lowest lethal concentration (LC_Lo_) of the HCl vapor, which results in the fatality of an individual human, has been reported to be 1300 ppm for 30-min exposure and 3000 ppm for 5-min exposure^36^. The RD_50_ (exposure concentration producing a 50% respiratory rate decrease) test is another standard method used as an indicator of the hazard of vapors. The 10-min RD_50_ value for mice has been reported to be 309 ppm^37^. Meanwhile, a permissible exposure limit for 8-hour time-weighted average (TWA-PEL) for HCl vapor in working spaces is 5 ppm^38^. Safety limits are very low compared to lethal concentrations because they consider long-time exposure over many hours. To sense the HCl vapor, sodium fluorescein is suitable since it has strong pH dependence in fluorescence and absorption due to the occurrence of protolytic reactions in the excited state; therefore, HCl and other strong acids can induce cation species that reduce the quantum yield and the blue-shift of the absorption peak^39^. Figure 2a shows absorption and emission spectra of sodium fluorescein doped silk films before (solid) and after (dotted) exposure to the HCl vapor. While the absorption spectrum was blue-shifted, the intensity of fluorescence decreased at the same wavelength. Along with the deteriorated fluorescence (usable for the fluorescent chemosensing), the color change of the FSN mat based on the shift of the absorption peak can be a good indicator for HCl detection.

The FSNs were exposed to vaporized HCl at different concentrations (300, 1300, and 3000 ppm). Figure 2b shows changes of the external color and fluorescence of the FSNs exposed to 300 ppm HCl vapor (10-min RD_50_ value). On increasing the exposure time, the fluorescent color was shifted from light-green (λ_peak_ = 535 nm) to dark-green (λ_peak_ = 510 nm), while the external color under daylight irradiation changed from light-yellow to light-green. Moreover, Fig. 2c shows fluorescent colors of the FSNs when they were exposed for 2 seconds at various concentrations. Increasing the concentration of HCl vapor results in a faster and clearer response.

Changes in photoluminescence and reaction rates at lethal concentrations of RD_50_ (300 ppm, for mice), LC_LO,30 min_ (1300 ppm, for humans) and LC_LO,5 min_ (3000 ppm, for humans) were compared in Fig. 2d–f. It is noteworthy that, for all concentrations, FSNs reveal immediate decaying in fluorescence. The elapsed time to deteriorate the fluorescence fully was just 18 seconds (300 ppm), 4 seconds (1300 ppm), and 2 seconds (3000 ppm). The fluorescence decay of the FSN was 16 times faster than that of the fluorescent silk film with the same compositions (Supplementary Fig. S2). This proves that the FSNs promise high sensitivities for detection of toxic vapors due to their high surface-to-volume ratio. In addition, we investigated responses to low concentrations. Supplementary Fig. S3 shows fluorescence spectra for the FSN chemosensors after 5-min exposure to HCl vapor with concentrations of 0, 5, 10, and 100 ppm. It could be seen that, even at a low concentration of several ppm, FSN sensors exhibited detectable signals within a few minutes, which is enough time to take safety measures. It is noteworthy that the silk fibroin could be a reliable and chemically stable matrix for chemosensing applications^35^ and the nanostructured silk showed no damage under the HCl vapor exposure (Supplementary Fig. S4). In addition, fatal hydrofluoric acid (HF) vapor could be also detected using the same FSN sensor (Supplementary Fig. S5). Besides acid vapors, the sphere of sensing applications can be expanded by introducing proper probe dyes into FSN platforms^40, 41^.

**Figure 3:**
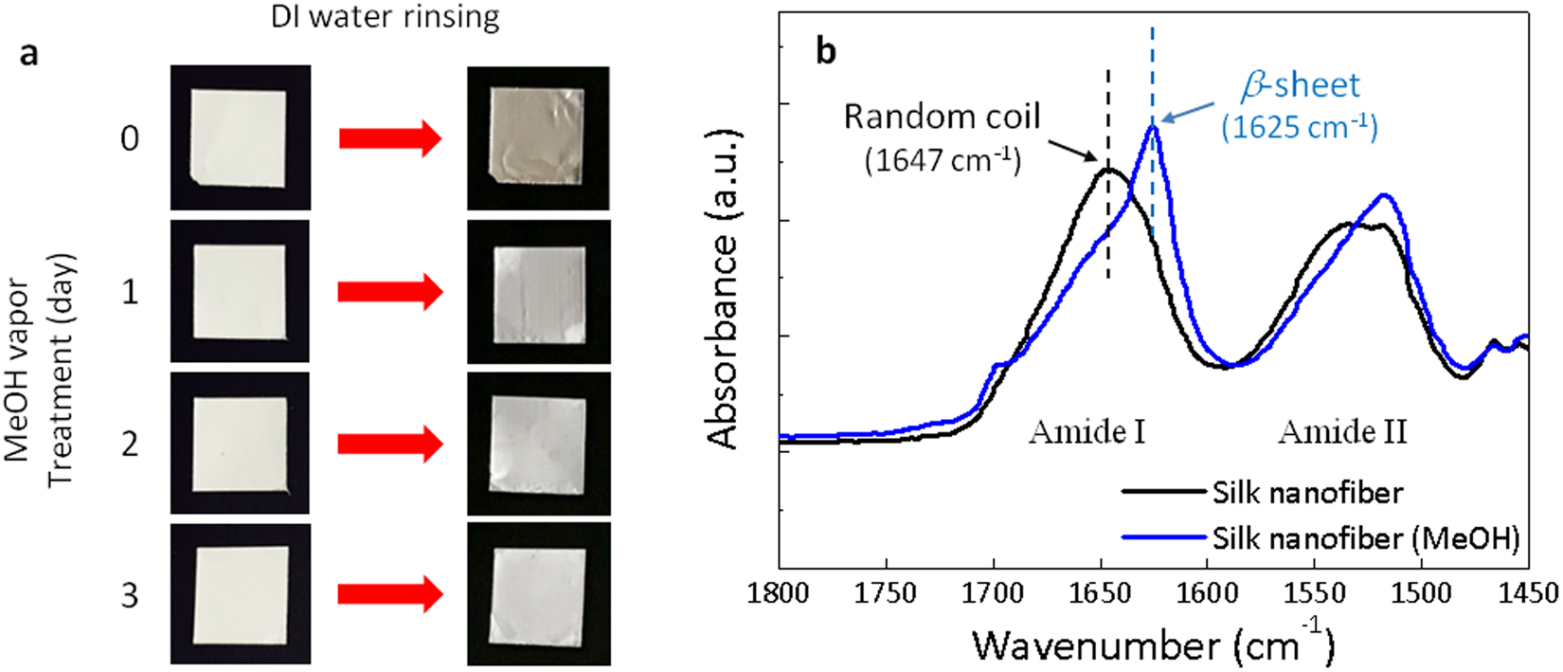
Solubility of the generated FSNs in water. (**a**) Effects of methanol vapor treatments on water solubility of FSNs. (**b**) FT-IR absorbance spectra of FSNs before (black line) and after (blue line) 3 days of methanol treatment. Amide I region indicates the increase of *β*-sheets, and decrease of random coils during methanol vapor treatment.

Solubility of the regenerated silk in water depends on the crystallinity of the silk molecules and can be a criterion to determine the lifespan of physically transient silk devices^42^. As shown in Fig. 3a, freshly generated FSNs from the electrospinning process easily dissolve in water due to lack of crystallinity. The methanol vapor treatment is a well-known method of increasing the crystallinity, a secondary structure of silk (*β*-sheet)^20^. The longer the methanol vapor treatment was performed, the more FSNs remained on the aluminum foil after rinsing with water (Fig. 3a). This polymorphic transition that correlates to water solubility was verified by means of Fourier-transform infrared (FT-IR) spectroscopy. Non-treated FSN showed a resonance absorbance centered at 1647 cm^−1^, indicating the presence of the typical random coil organization of the amorphous protein. After treating the FSN with methanol vapor for 3 days, a sharp peak appeared at 1625 cm^−1^, which was a clear evidence of the *β*-sheet formation of the silk fibroin^42^. In addition, the stability of the FSN membranes was investigated since the non-crystallized silk fibroin is water-soluble and therefore weak to moisture. To obtain a uniform silk nanofiber network, it is desirable that the humidity of the electrospinning chamber is kept within 30% RH^43^. The relative humidity of the laboratory has been maintained at less than 30% RH, where the FSNs can be very stable over a week. We investigated the appropriate relative humidity to utilize the FSNs practically. As shown in Supplementary Fig. S6, the nanofiber network was crushed when exposed to high humidity over 60% RH, which meant the reduction of the surface-to-volume ratio and its sensing performance. However, note that the low stability of the non-crystallized FSN to water can be improved by controlling the crystallinity.

**Figure 4:**
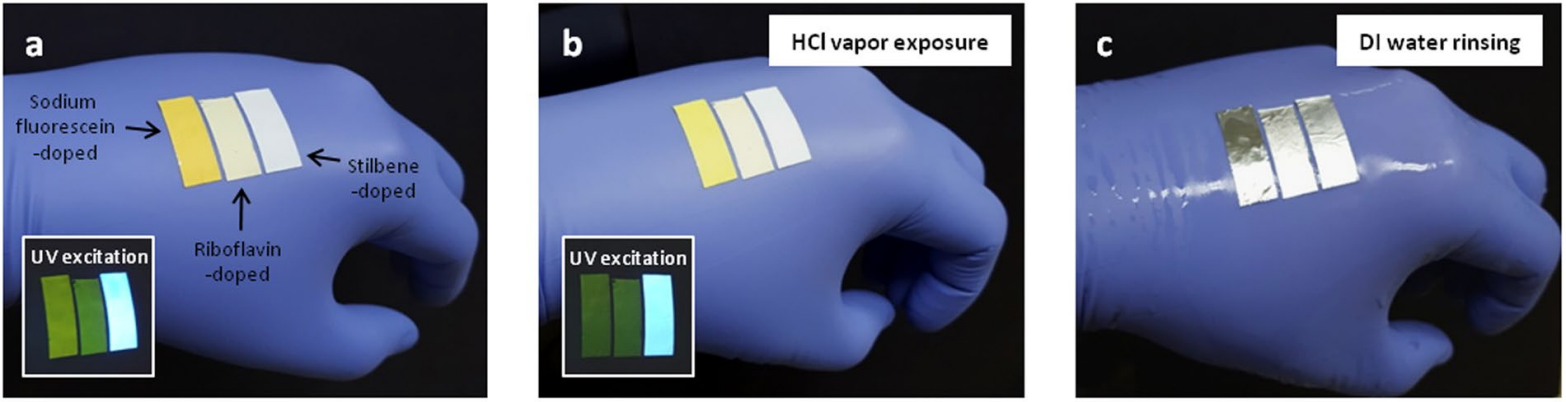
FSNs as disposable hazard indicators. (**a**) Rectangular pieces of FSNs attached on an experimental glove. (**b**) FSNs exposed to HCl fume. FSNs doped with sodium fluorescein only react to HCl exposure, while other FSNs doped with riboflavin or stilbene remain unchanged. (**c**) DI water rinsing to remove FSNs.

Additional favorable traits of FSNs spun on aluminum foil are flexibility, conformability, and easy processing. Hence, we applied the FSNs as a hazard indicator by easily attaching them to work clothes or protective equipment. As shown in Fig. 4a, rectangular pieces of FSNs were attached on an experimental glove as an HCl indicator (doped with sodium fluorescein, riboflavin, and stilbene, from left to right). Note that all organic dyes embedded in the nanofibers are biologically usable. After exposing FSNs to the HCl fume for 1 minute, only sodium fluorescein doped FSN exhibited a change in its fluorescent property as we already proved, while other FSNs doped with riboflavin and stilbene showed no response to HCl vapor (Fig. 4b). An interesting feature is that the used FSNs can be removed with no pollution upon water rinsing since FSNs are made of environmentally harmless substances (Fig. 4c). Furthermore, a free standing FSN mat could be conformally laid on skin and used as a thin, disposable skin-type sensor (Supplementary Fig. S7).

**Figure 5:**
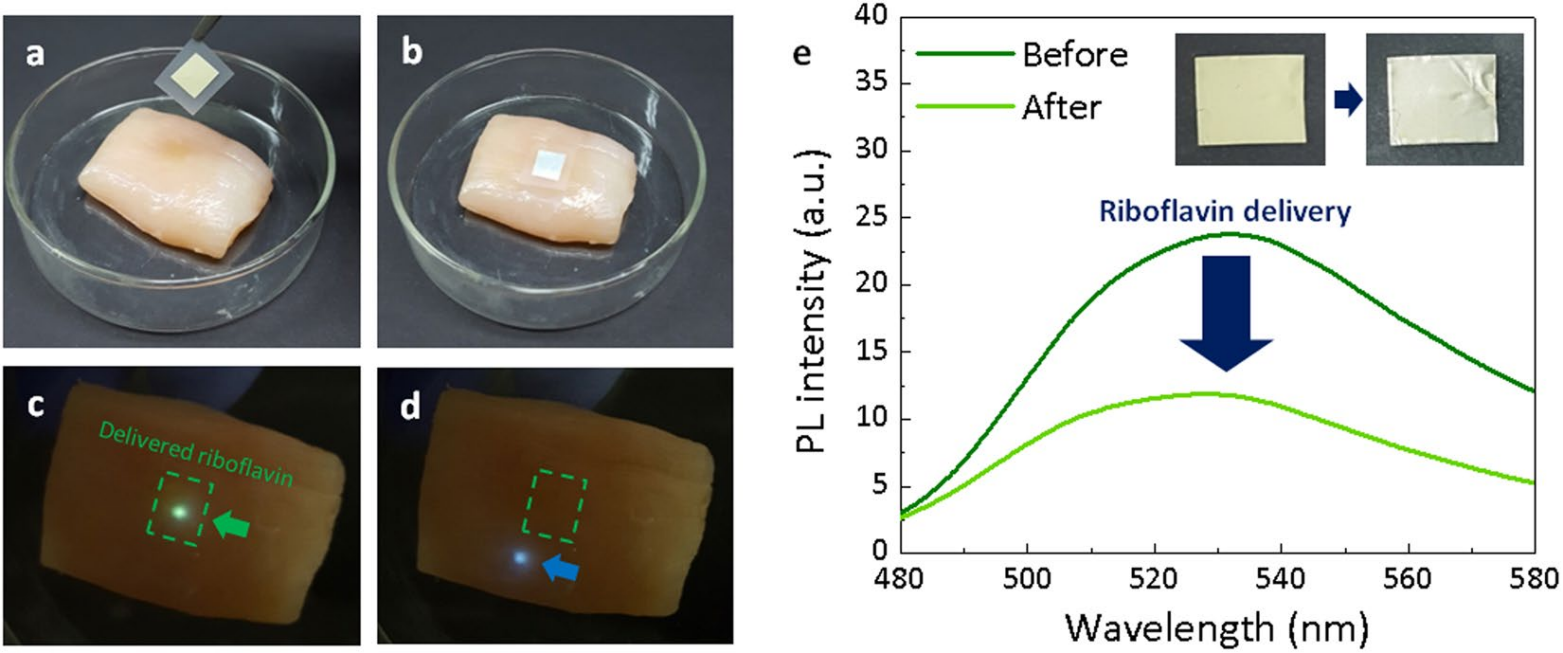
Simple model for a nutrient delivery system. (**a**,**b**) A riboflavin-doped FSN mat is placed on the surface of a raw chicken breast (as a biological tissue). (**c**,**d**) Confirmation of riboflavin delivery to the skin. The region on which the FSN mat was placed is marked with a green dotted square. Inside the green dotted square, delivered riboflavin responds to UV excitation (325-nm He-Cd laser) and shows green fluorescence. (**e**) Photoluminescence spectra of FSNs doped with riboflavin before and after vitamin delivery. Inset shows the change of the external color of the FSN mat.

Nanofibers that can transfer embedded drug to a biological tissue due to high surface-to-volume ratio have been widely used in delivery systems for tissue scaffolds, anti-bacterial sheets, and anti-cancer^44^. Biocompatibility of silk fibroin makes it possible for implanting and resorbing FSNs into humans. In addition, fluorescence of the doped functional material is useful for imaging and qualify its delivery. As a proof-of-the-concept, we created a model for a nutraceutical delivery system to skin using a riboflavin-doped FSN. Riboflavin is known as vitamin B_2_, a nutrient, and known to be effective in skin care for controlling acne and sebum. As shown in Fig. 5a,b, a riboflavin-doped FSN mat was placed on the surface of a biological tissue (chicken breast). To stabilize the network of the silk matrix while the absorbed water molecules extracted riboflavin, the FSN was crystallized using methanol vapor in advance. For 2 seconds, the riboflavin embedded in the mat was dispersed to the tissue surface, and then the FSN mat was detached from the tissue. We could trace the delivered riboflavin by observing its fluorescence under UV excitation. Figure 5c and d show photographs of the biological tissue excited by the UV light source. We optically pumped regions where riboflavin was delivered (Fig. 5c) and was not (Fig. 5d), and observed that green fluorescence of riboflavin occurred in the riboflavin-delivered region. By comparing the intensity of fluorescence of the FSN between before and after the delivery experiment, the amount of the delivered riboflavin can be estimated quantitatively, as shown in Fig. 5e. The fluorescence of the riboflavin silk mat was remarkably decreased by 60% during the 2 seconds of delivery, and the appearance also became closer to white as riboflavin dyes escaped.

## Conclusion

In conclusion, we demonstrated biocompatible, eco-friendly, and cost-effective FSN mats by means of the electrospinning method and their uses for a physically transient chemosensor and nutrient deliverer. Uniform and large area FSN mats were successfully prepared using water soluble organic dyes. Especially, sodium fluorescein doped FSN was instantly responsive and highly sensitive to HCl vapor. A few ppm of HCl vapor was enough to induce detectable change in fluorescence. We could program the lifespan of the FSN by controlling the crystallinity of silk matrix that would be useful for realizing physical transient and functional FSN devices. In addition to flexibility and easy processing, favorable traits of silk fibroin would be applicable to a disposable and skin-type hazard indicator that could be attached on clothing or protective gears. Delivering riboflavin from a FSN mat to a biological tissue could be traced by the fluorescence measurement. Our achievements strongly suggest the application of FSN mats as an outstanding sensing material in environmental and biological sciences.

## Methods

### Preparation of aqueous silk solution

Cocoons of *Bombyx mori* caterpillars were boiled for 30 minutes in an aqueous solution of 0.02 M Na_2_CO_3_ to remove sericin proteins. Then extracted silk fibroin was rinsed with distilled water for 20 minutes, 3 times and dried for a day. After drying the silk fibroin was dissolved in 9.3 M LiBr solution and incubated in oven at 60 °C for 4 hours. This solution was dialyzed against distilled water using a dialysis cassette (Cellu-Sep T1, Membrane Filtration Products, MWCO 3.5 K) at room temperature for 48 hours. The obtained solution was centrifuged for 20 minutes at −1 °C with 9,000 rpm, twice to remove impurities. The final concentration of silk fibroin aqueous solution was approximately 8 wt%.

### Electrospinning process for generation of fluorescent silk nanofibers

The addition of poly(ethylene oxide) (PEO, M_v_ ~900,000, Sigma-Aldrich) to silk solutions generated a viscosity and surface tension suitable for electrospinning. The base solution was made by mixing the 5 wt% PEO solution and prepared 8 wt% silk solution at a ratio of 1:1. Then, 10 mM of active dye was mixed with the base solution. In our experiments, 4 types of dye, including stilbene (Stilbene 420, Exciton), sodium fluorescein (Fluorescein sodium salt, Sigma-Aldrich), riboflavin (Riboflavin 5′-monophosphate sodium salt hydrate, Sigma-Aldrich), and rhodamine B (Rhodamine B, Sigma-Aldrich), were used. The prepared silk bio-ink was injected in the syringe and mounted on the electrospinning machine. An aluminum foil was used as the collection screen. The distance between the tip and the collector was 20 cm, and flow rate of the bio-ink was set to 10 μL/min. The 10 kV of electric potential was applied between 21 G nozzle tip and collector during electrospinning time between 30 minutes and 2 hours. Finally, the generated FSNs had a uniform diameter of 300–350 nm.

### Preparation of vaporized HCl and concentration control

To prepare the HCl vapor environment on a ppm scale, the liquid HCl was dropped with a micropipette in a beaker. After sealing the beaker, we waited for 2 hours at room temperature to completely vaporize the liquid HCl. For a concentration of 1 ppm (v/v), the ratio of the volume of the HCl vapor to the beaker volume was adjusted to 1 μL/L. By evaporating 1.63 μg of liquid HCl, 1 μL of HCl vapor could be obtained, which was estimated from the molar mass to molar volume ratio at room temperature.

### Optical measurements of silk nanofibers

Fluorescent spectra of FSNs were measured using a vis/near-infrared spectrometer (iHR-320, HORIBA Jobin Yvon), and the FSNs were optically pumped by the 325-nm He-Cd continuous-wave (CW) laser. In order to excite the FSNs over a large area (Fig. 1c,d), the pumping spot of the laser was expanded using an optical lens with a focal length of 5 cm. To observe the silk nanofibrous structures and estimate the diameters of fibers, the SEM images were taken (S-4800, Hitachi). To analyze the secondary structure of silk protein, FT-IR measurements were performed (Nicolet iS50, Thermo).

## Electronic supplementary material

Supporting Information
