# Supplementary material for: Colored and fluorescent nanofibrous silk as a physically transient chemosensor and vitamin deliverer
Source: Sci Rep. 2017 Jul 14;7:5448. doi: 10.1038/s41598-017-05842-8 (PMC5511142; doi:10.1038/s41598-017-05842-8)
Supplement: Supplementary file 1 — Supporting Information [file 41598_2017_5842_MOESM1_ESM.doc]

**Supplementary Information**

Colored and fluorescent nanofibrous silk as a physically transient chemosensor and vitamin deliverer

Kyungtaek Min^1,†^, Sookyoung Kim^1,†^, Chang Gun Kim^2^ & Sunghwan Kim^1,3,*^

^1^Department of Energy Systems Research, Ajou University, Suwon 16499, Republic of Korea

^2^Immune-Network Pioneer Research Center, School of Medicine, Ajou University, Suwon 16499, Republic of Korea

^3^Department of Physics, Ajou University, Suwon 16499, Republic of Korea

^*^[To whom correspondence should be addressed. E-mail: sunghwankim@ajou.ac.kr](mailto:sunghwankim@ajou.ac.kr)

^†^These authors contributed equally to this work.

**FSNs doped with various organic dyes**


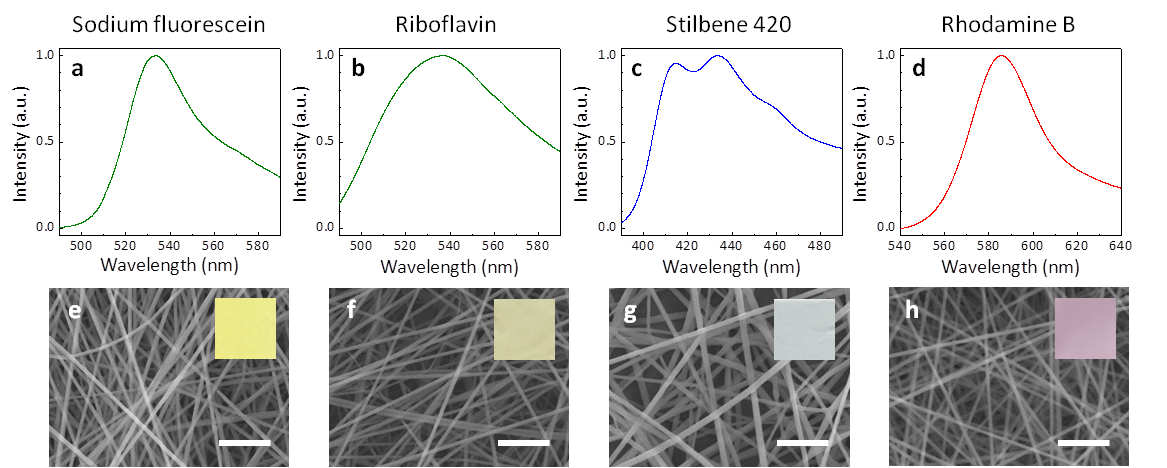


**Figure S1.** Fluorescence spectra of FSNs doped with (a) sodium fluorescein, (b) riboflavin, (c) stilbene 420, and (d) rhodamine B, which emission peaks occur at λ_peak_ = 535, 540, 435, and 585 nm, respectively. (e-h) SEM images of the FSNs. Insets represent external colors of each FSN mats. Scale bars represent 5 μm.

**A HCl vapor sensor utilized by a thin flat silk film doped with sodium fluorescein**

**Figure S2.** Fluorescence spectra of a thin fluorescent silk film. The florescent silk film was prepared by spin-coating the sodium fluorescein-doped silk solution on silicon substrate. The thickness of the film was around 300 nm. The elapsed time to deteriorate the fluorescence fully was 5 min at the concentration of 300 ppm.

**HCl vapor sensing experiment (low concentration)**

**
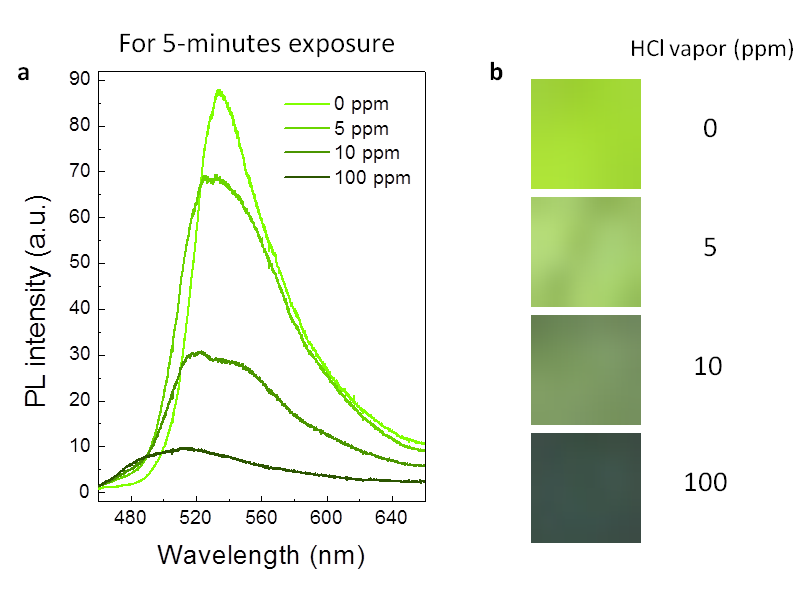
**

**Figure S3.** (a) Fluorescence spectra of the FSNs doped with sodium fluorescein after 5-min exposure to HCl vapor with concentration of 0, 5, 10 and 100 ppm. (b) Concentration-dependent fluorescence changes by exposure to HCl vapor for 5 min.

**SEM images of FSNs before and after exposure to the HCl vapor**

**Figure S4.** SEM images of FSNs (a) before and (b) after exposure to the HCl vapor. Silk nanofibers showed no influence on exposure to the HCl vapor. Inset shows the change of the external color of the FSN mat. Scale bars represent 2 μm.

**Hydrofluric acid (HF) reaction test**


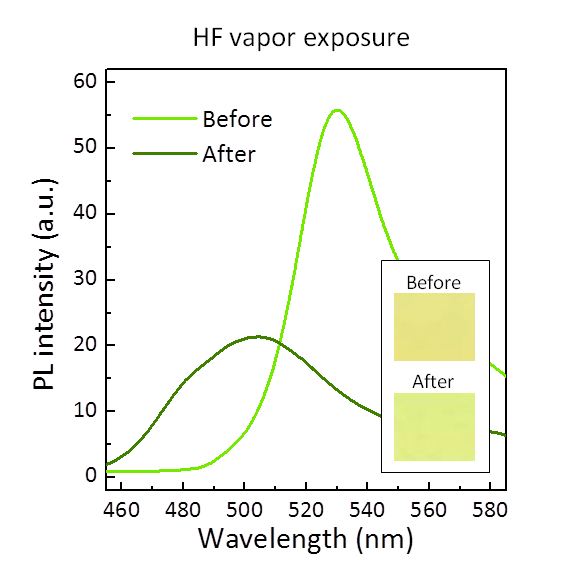


**Figure S5.** Fluorescence spectra of the FSNs doped with sodium fluorescein, before (black line) and after (red line) exposure to HF fume. Inset displays change of the external color of FSNs.

**Stability of FSNs against water in air**

**Figure S6.** SEM images of the FSNs exposed to moisture with various humidity values over an hour. The FSNs were stably maintained at (a) 40% RH and (b) 60% RH, whereas the fiber networks were crushed at (c) 70% RH and (d) 80% RH. Although the fluorescence (insets) before and after exposure to moisture is not significantly changed, performances of the FSN sensors are not reliable at high humidity above 60% RH. Scale bars represent 2 μm.

**Disposable skin-type FSN mat**


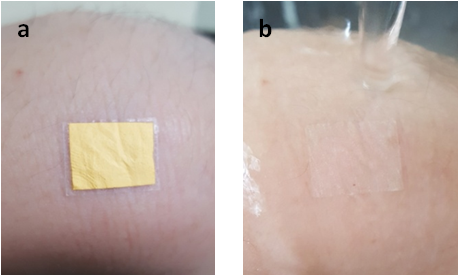


**Figure S7.** (a) A FSN mat doped with sodium fluorescein can be conformally attached on the skin. (b) The FSN mat is removed readily by DI water rinsing.
